# Laboratory Assessment of an In-Place Inclinometer Chain for Structural and Geotechnical Monitoring

**DOI:** 10.3390/s23208379

**Published:** 2023-10-10

**Authors:** Francesco Freddi, Lorenzo Mingazzi, Emilio Pozzi, Nicola Aresi

**Affiliations:** 1 Department of Engineering and Architecture, Università degli Studi di Parma, 43124 Parma, Italy; lorenzo.mingazzi@unipr.it; 2 SISGEO SRL, Via F. Serpero 4/F1, 20060 Masate, Italy; emilio.pozzi@sisgeo.com (E.P.); nicola.aresi@sisgeo.com (N.A.)

**Keywords:** in-place inclinometers, inclinometer chain, tilt sensors, landslides, monitoring systems, ground deformation

## Abstract

The necessity of early warning systems to ensure people’s safety requires the usage of real-time monitoring instrumentation. To meet the required real-time monitoring performance, in-place inclinometer systems represent one of the most common solutions to obtain accurate measures over time. This paper presents the results of a laboratory tests campaign performed on the prototypes and preproduction samples of an in-place inclinometer chain for structural and geotechnical monitoring applications. First, each element sensor has been calibrated to reach a proper level of measure accuracy. Eventually, laboratory tests are carried out on both a single instrument (element) and on the complete measurement chain (system). The adopted centering device, obtained as a combination of a Cardan joint and four spring plungers avoids bending of elements by preventing fictitious displacement measurements and permits the creation of a kinematic chain that accommodates the displacements of a grooveless tube. A specially designed and constructed test set-up that permits assigning a movement to each node has been employed to test a specifically designed centering device and check the system stability over time. Different scenarios have been investigated to determine the accuracy and repeatability of the measures in replicating real cases. The results demonstrated the necessity of validating a measurement chain by analyzing its overall behavior and not limiting the study on the performances of a single element.

## 1. Introduction

Geotechnical instrumentation plays a crucial role in assessing the performance and behavior of structures in the built environment. Moreover, the capability to monitor soil, ground, and structural movements over time represents a fundamental aspect in the prevention and containment of casualties. In fact, early identification of potentially dangerous deformations is a key factor in significantly minimizing losses [1,2,3]. A wide variety of instruments can be employed, such as GPS/GNSS to track three-dimensional movements of ground surfaces [4,5], inclinometers to evaluate ground surface and sub-surface displacements as well as deformations in structural elements [6,7,8], extensometers to measure settlements in excavation, foundations and embankments [9], or tilt meters [10]. Combining different monitoring strategies permits engineers to obtain an exhaustive representation of the actual condition of the analyzed slope or structure, resulting in a more detailed description of displacements and deformations affecting the monitored object [2,11,12,13].

In fact, characterization of slopes behavior over time cannot be limited to the analysis of ground movements but also requires information on subsurface evolution and deformation. Similarly, for structural applications, certainty of adequate safe working conditions for retaining walls, diaphragms, and embankments can be achieved if detailed information on the deformations of the whole structure is available [14,15].

To this extent, inclinometers and inclinometer chains represent the most common and accurate methods used in subsoil and structural health monitoring [1,6,15,16]. They provide accurate measurements on underground displacements, thus providing valuable information on the eventuality of possible dangerous ground movements such as landslides, subsidence, or rupture of a structural element [17]. Using these data, engineers can identify any potential issues and take appropriate measures to prevent accidents and ensure the safety of the construction workers and the structures being built. These instruments are therefore essential for detecting early signs of deformation or instability and providing real-time data to help engineers and researchers make informed decisions [18].

While providing accurate measurements, the use of conventional inclinometers is time consuming and can hardly meet the requirements of real-time monitoring [19]. To achieve this required monitoring performance, in-place inclinometer (IPI) systems have been developed [20], representing an essential tool for continuous monitoring of the deformation of structures, retaining walls, embankments, and slopes in structural and geotechnical engineering [20,21,22].

IPI systems can be installed at the desired depths to monitor the movements in near real time using automated data acquisition equipment, providing critical information on the magnitude, direction, and rate of movement. IPI consists of a series of beam sensor chains connected and placed inside an inclinometer casing, allowing the deformation curve to be generated by multiplying the tilt readings with their corresponding lengths [17]. This curve can then be used to assess any shifts and evaluate the structural stability of the monitored site.

The use of Micro Electro-Mechanical Systems (MEMS) has been introduced within IPI systems to provide a higher temporal measurement resolution as well as a reduction in manufacturing costs [23,24,25,26,27]. Commercially available instrumentation is varied: in some cases, an inclinometer chain is created to be inserted into grooved tubes [23,24,27] or flexible chains are housed and rest on the wall of tubes [26]. Despite their wide usage, the accuracy and reliability of IPI systems must be validated to ensure the quality of the data they produce [28,29]. Alternatively, distributed fiber optic sensors can be adopted for displacement measurements as illustrated in [30] where a detailed validation of 3DSensor is performed. This strategy seems very promising because it permits very fine measure points, high precision, and ease of installation. In addition, structural profiles derived from vertical displacements can be obtained by hydraulic sensor once that temperature is compensated as illustrated in [14].

The present paper illustrates the main features of the so-called MD-Profile developed by SISGEO and Patented product (N. 102021000011177) [31] as well as their validation procedures. The MD-Profile employs a specifically designed centering device that allows the usage of grooveless tubes, leading to an easier installation procedure for the instrumentation as no preferred orientation of the system is needed. Additionally, tube spiraling-related problems are also avoided.

First, calibration of the MEMS sensor is performed using a high-precision calibration bench which ensures level of accuracy compliant to the current standards. Eventually, via the usage of a specially designed and constructed test setup, an extensive laboratory campaign has been performed to investigate the IPI system accuracy. Initially, the single elements (instruments) that make up the inclinometric chain (system) have been tested to determine the accuracy and reliability of a single instrument to provide accurate measurements. However, the analysis of the single instrument fails to correctly characterize the accuracy of the complete system as inaccuracies in the measures may rise due to the interactions between elements. Nonetheless, very few reports of the characterization and testing of the whole chain are present in the literature [14,30]. To this extent, a series of tests involving the whole inclinometric chain have been specifically developed aiming to replicate deformed configurations representing various failure conditions in soil or structural elements.

This paper is structured as follows. In Section 2, the IPI-system mechanical and electrical components are presented. Section 3 presents the calibration and tests’ methods used to validate the accuracy of the instrumentation. Eventually, Section 4 reports the test results, and their discussion in Section 5 completes the paper before the conclusions of Section 6.

## 2. Material

**Figure 1:**
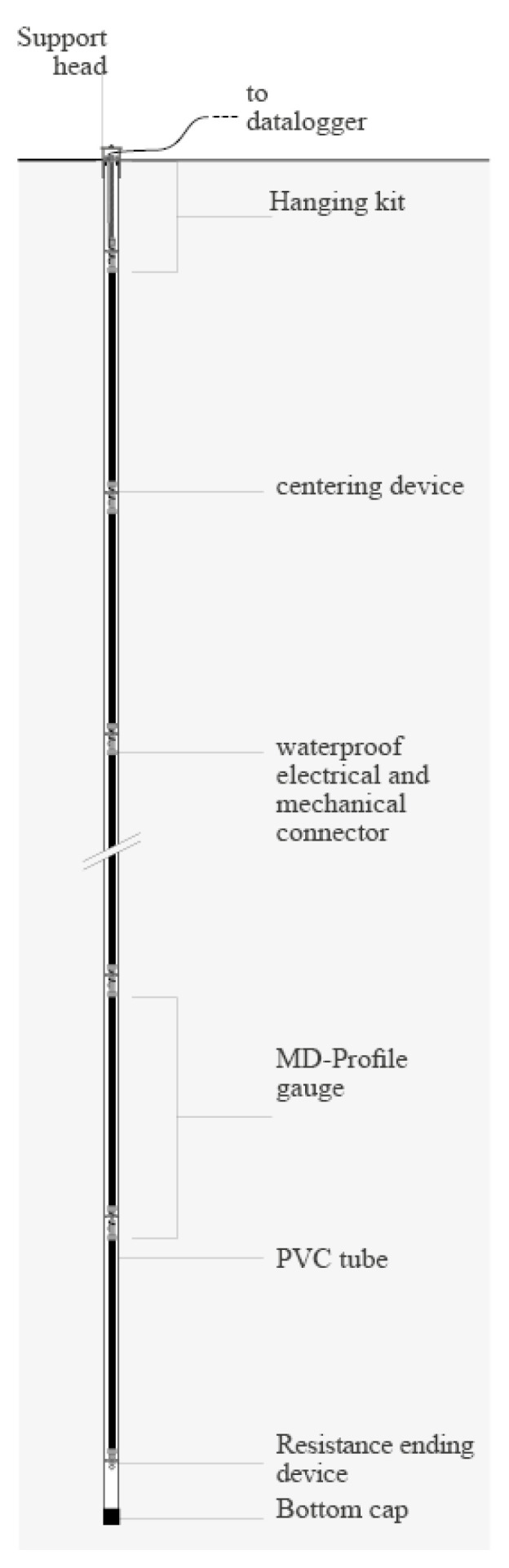
Vertical in-place inclinometer system configuration.

**Figure 2:**
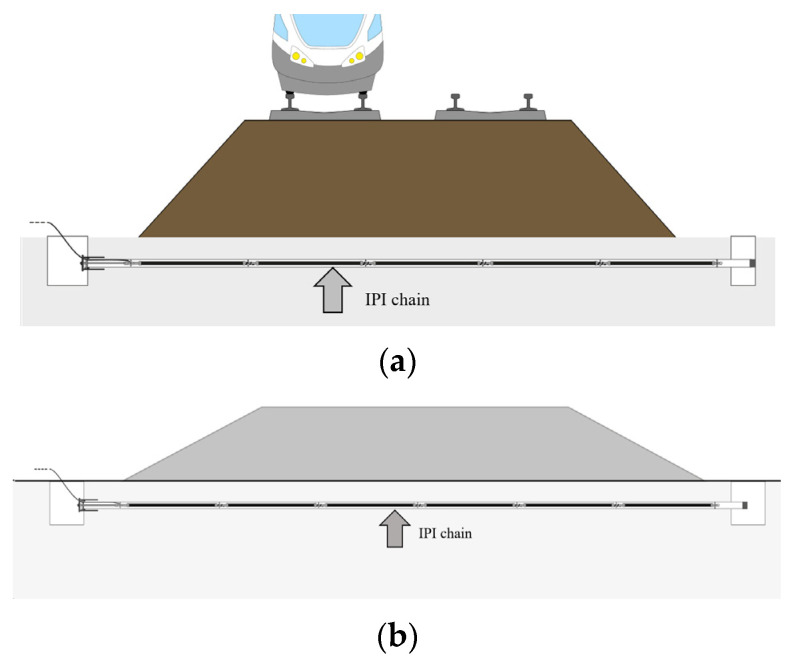
Horizontal in-place inclinometers: (**a**) application to railway embankment monitoring; (**b**) horizontal in-place inclinometer system configuration.

**Figure 3:**
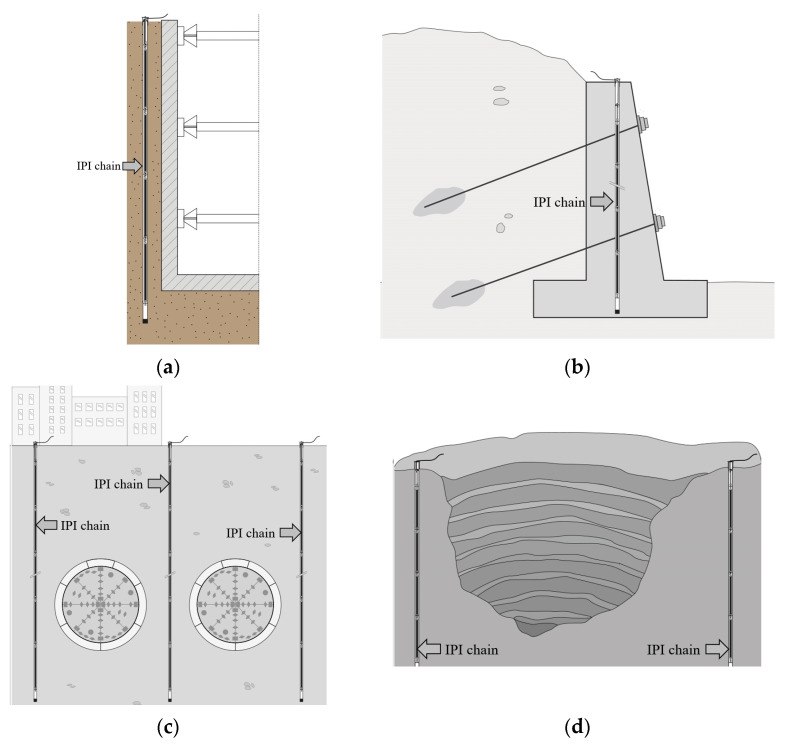
Application of vertical in-place inclinometers: (**a**) deep excavation; (**b**) retaining walls; (**c**) tunneling; (**d**) open pit mines.

MD-Profile is an inclinometric chain (Figure 1) suitable for geotechnical and structural applications where vertical or horizontal accurate profiling (cumulative displacement along the same line) is required. Possible applications such as monitoring embankments, landslides, rockfalls, tunnels and excavations are illustrated in Figure 2 and Figure 3. This measuring system is particularly effective in providing early warning of potential failures thus enabling rapid responses to mitigate risks. The high-precision measurements of the system can also be useful in validating numerical models of soil and rock behavior, improving the understanding of these materials’ properties. The basic principle of operation is the utilization of MEMS tilt sensors to make inclination measurements over segments of a borehole drilled into the structure being studied.

In particular, the MD-Profile is configured to measure relative deformations (i.e., with respect to a zero measurement) in different directions, including horizontal and vertical movements. Measured data are recorded via a MODBUS RTU datalogger [32], sent to a central monitoring station and eventually analyzed and interpreted to provide real-time information about the deformation of the object being monitored.

### 2.1. Mechanical Components

**Figure 4:**
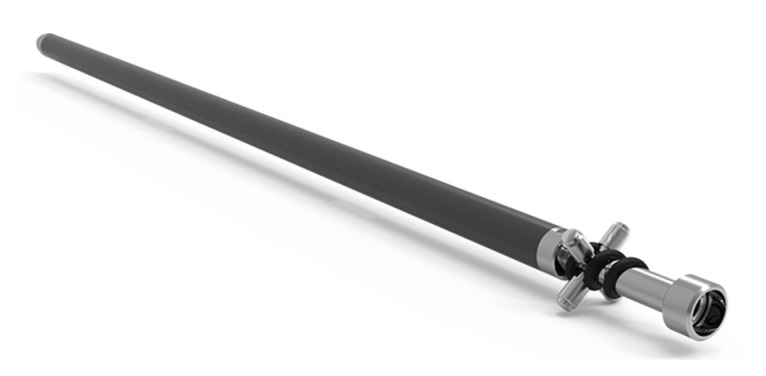
Single inclinometer instrument.

MD-Profile instruments (Figure 4) are designed to be placed within grooveless flush tubes of limited diameter (1.5 ÷ 2 inches). The usage of non-directional tubes allows an easier system installation process, as alignment of the instrument with respect to a preferred direction is not required. Additionally, spiraling-related problems can be avoided.

Different instrument lengths are available, ranging from 0.5 m up to 2 m, based on the resolution required by the application. Thanks to the use of lightweight materials such as carbon fiber, the weight of each instrument is limited and ranges from 0.65 kg for the 0.5 m instrument up to 1.05 kg for the 2 m instrument, thus facilitating transport and installation operations.

**Figure 5:**
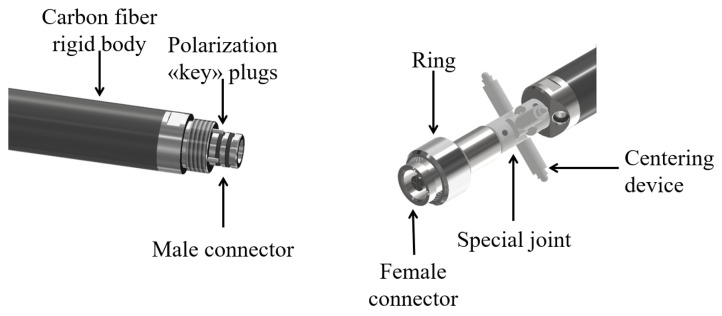
Components of the connection.

Each instrument is mechanically and electrically linked to one another through connectors in a linear bus topology that avoids the necessity of external electrical connectors. This connection, however, requires the correct alignment of two subsequent instruments during the assembly of the system to avoid damaging the pins that are transferring the electrical signal throughout the inclinometer chain. The components of the connection between two subsequent instruments of the chain are reported in Figure 5.

**Figure 6:**
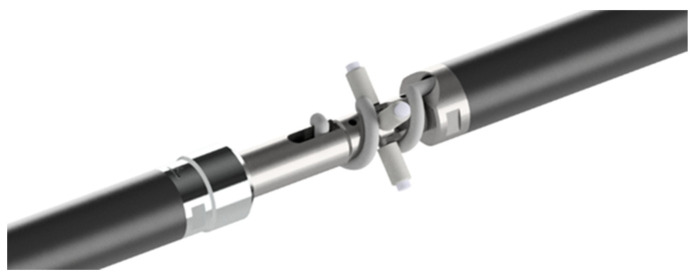
Details of the connection between the two inclinometers.

The position of the whole chain inside the tube is kept centered by means of a centering device that provides long-term stability avoiding unwanted movements of the nearby instruments. The device is composed of a Cardan joint and four spring plungers and is located exactly at the junction between two adjacent instruments (Figure 6). This connection does not create any bending in the elements nor interferences on the nearby probes along the chain. Additional details will be given in Section 4.

### 2.2. Electronic Components

Each MD-Profile is equipped with an electronic board with a microcontroller able to manage the MEMS appropriately and to process the raw measurement data, applying special calibration algorithms via edge computing. The electronic board has been designed to have an extended operating temperature range from −30 to +70 °C and a reduced consumption of 3 mA at 24 Vdc and 5 mA at 12 Vdc. Additionally, each electronic board is equipped with sensors for internal diagnostics at each measuring point recording temperature and voltage supply. The sensing element is a MEMS accelerometer that measures the acceleration due to gravity on 1, 2, or 3 axes [33]. In this application, the dynamic effects are negligible. The MEMS is mounted on a dedicated Printed Circuit Board (PCB) and a microcontroller is used to configure and read data from the MEMS.

Inclination data recorded from the instruments are provided directly in engineering units with a default output of sine-angle.

Data transmission takes place via RS-485; therefore, only one connection cable is required between the master and the MD-Profile chain. Cable lengths of up to 1000 m are easily reachable. The measurements are then made available through a standard digital protocol (MODBUS RTU).

Any master, datalogger, or readout compatible with the RS-485 and Modbus RTU protocols can be adopted as a logger. A complete and transparent set of data is recorded and given to the end user from the MD-Profile system.

## 3. Calibration Methods

### 3.1. Calibration of the Inclinometric Sensor

The raw data provided by MEMS are not accurate enough for most inclinometric applications. Therefore, the calibration process plays a crucial role [29]. By means of special calibration benches, the sensor is subjected to known inclinations and the recorded values are used to calculate the calibration parameters which will then be applied to the measurements.

**Figure 7:**
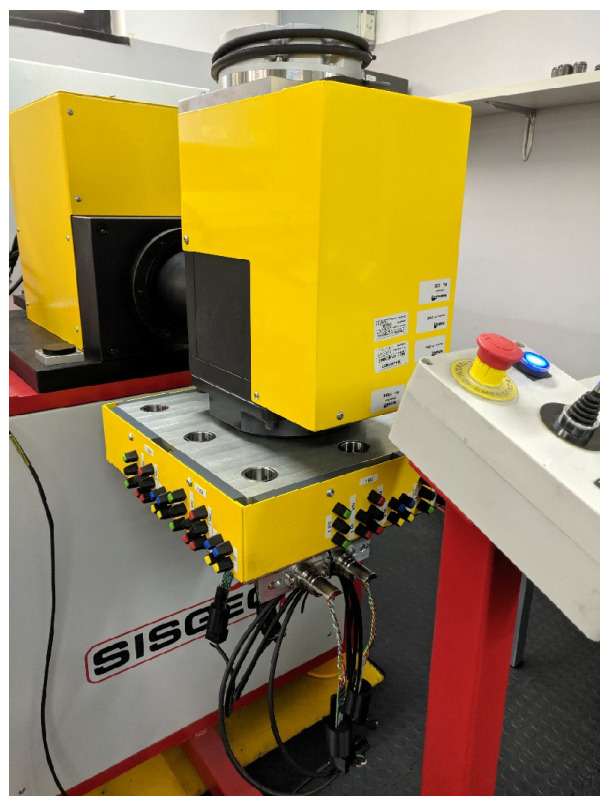
Calibration bench for the inclinometer sensors.

Each sensor is individually calibrated in the SISGEO laboratory following high-level metrological procedures. The procedure is automatically performed through specially developed benches (Figure 7) and periodically verified through certified reference standards ISO 10012:2003 [34]. The calibration, in addition to the linearization, reduces the cross-correlation between the axes, the mechanical offset, and the electrical offset. After the calibration process, a detailed report is produced for each gauge.

### 3.2. Calibration of the Inclinometric Chain

In order to provide unaltered measurement values from the inclinometer chain, two fundamental aspects must be guaranteed:

1. The interaction with the inclinometric casing must be punctual.
2. The chain must be a kinematic mechanism completely free of inflections.

To achieve this dual result, the rods must be connected via joints, and the interaction forces must act on the hinges themselves. Positional constraints that are not optimal result in the appearance of inflections in the rods which, although limited, lead to false displacement readings. In addition to accuracy, inclinometric chains must guarantee measurement repeatability and, above all, stability. Operationally, it is simple to test the performance of the electronics and a single element. However, this procedure is not sufficient to be able to affirm that these performances can and must be transferred to the entire inclinometric chain. On the other hand, validating the behavior of a measurement chain is extremely complex. In fact, the interaction between the individual beams, the interaction between the probes and the inclinometric tube, and the installation operations undoubtedly alter or at least make the behavior of the inclinometric chain more complex and articulated. It is therefore necessary to provide test setups that reproduce, albeit limited in size and number of elements, the entire measurement chain. For this purpose, an ad hoc test setup has been developed that allows the reproduction of the main movements that the chain is required to reveal and stability tests to be performed.

The tests described here were performed at the Laboratory of Testing Materials of the Department of Engineering and Architecture of the University of Parma (UNIPR). The test apparatus, specifically designed to be used on a reinforced concrete wall located within the laboratory, has permitted the movement and control of fixed points placed in predetermined positions on the wall itself. Moreover, the use of a massive concrete wall ensures that the reference points of the test setup are fixed in the short and medium term. The main purpose of the test apparatus is to compare the values of imposed displacement with the measures obtained by the instrumentation produced by SISGEO thus allowing testing of the accuracy, precision, and repeatability after an initial check against stability over time.

**Figure 8:**
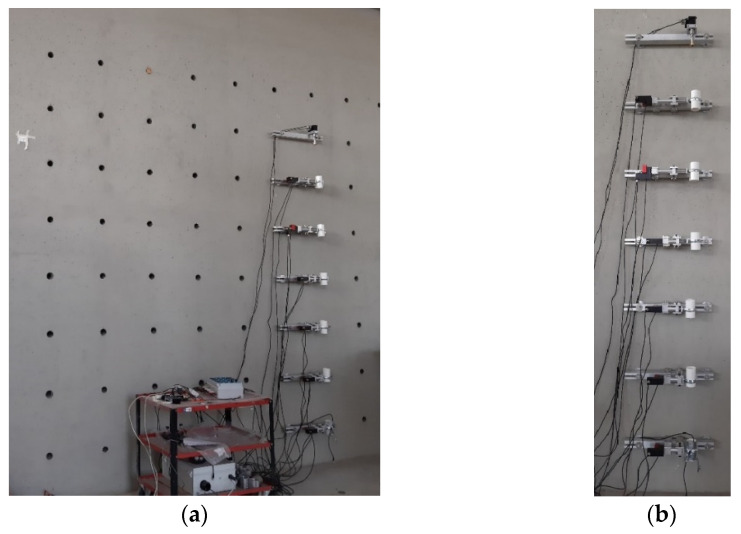
Sensor test setup: (**a**) concrete wall; (**b**) actuators setup used for inclinometer testing.

**Figure 9:**
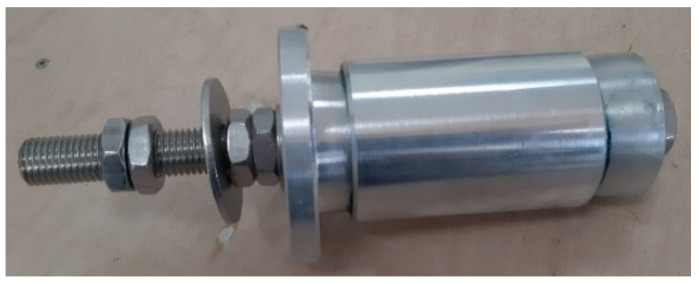
Expansion plug used to anchor the actuators to the concrete wall.

**Figure 10:**
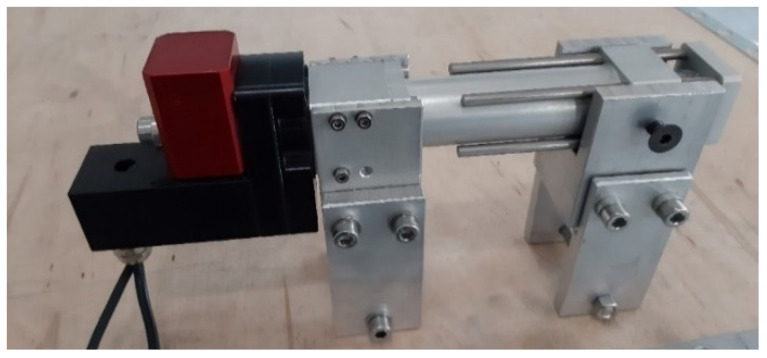
Actuator system that combines a 100 mm electric rotary actuator with a magnetic angular sensor. Fixing plates are installed on the actuator system to anchor it to the aluminum supporting beam.

The test configuration is now succinctly explained. The Laboratory of UNIPR’s concrete wall was utilized to install the testing apparatus. The concrete wall, depicted in Figure 8a, measures 10 m in length, 5 m in height, and 2 m in depth, and is outfitted with a grid of holes equally spaced at 0.5 m intervals. This wall is well suited for the testing objectives because it provides adaptability, convenient equipment access, and long-term stability. Figure 8b depicts the test arrangement, which comprises linear actuators responsible for the motion of the inclinometer tube sections. These actuators are fixed to aluminum beams, which are secured to the concrete wall through the custom-designed expansion plugs shown in Figure 9. The displacement is imposed using the Servo-OP produced by FIAMA [35], which is an electric rotary actuator. The rotation is then converted into linear movement via a linear actuator with a screw pitch of 4 mm, with either 50 or 100 mm stroke. The position is determined by measuring the rotation with a magnetic sensor mounted on the rotating shaft. The sensor has a resolution of 1000 impulses per revolution, which in the current setup is equivalent to 4 µm. The measuring system is precise to an accuracy up to 0.02 mm. Additionally, to ensure the durability of the geared motor and its connected components, the output shaft is equipped with a torque limiter that has a rating of up to 5 Nm. The operator can set and visualize the position and adjust the actuator’s speed through a control panel. A complete system of rotary and linear actuators is shown in Figure 10.

The probes are attached to the top beam of Figure 8b. This point serves a dual purpose: it fixes the end and enables the adjustment of the column’s orientation with respect to the imposed displacement by rotating the whole inclinometer chain along the longitudinal axis of the rods. Eventually, it is possible to impose a displacement in a predetermined direction. The setup permits the setting of multiple displacement configurations.

**Figure 11:**
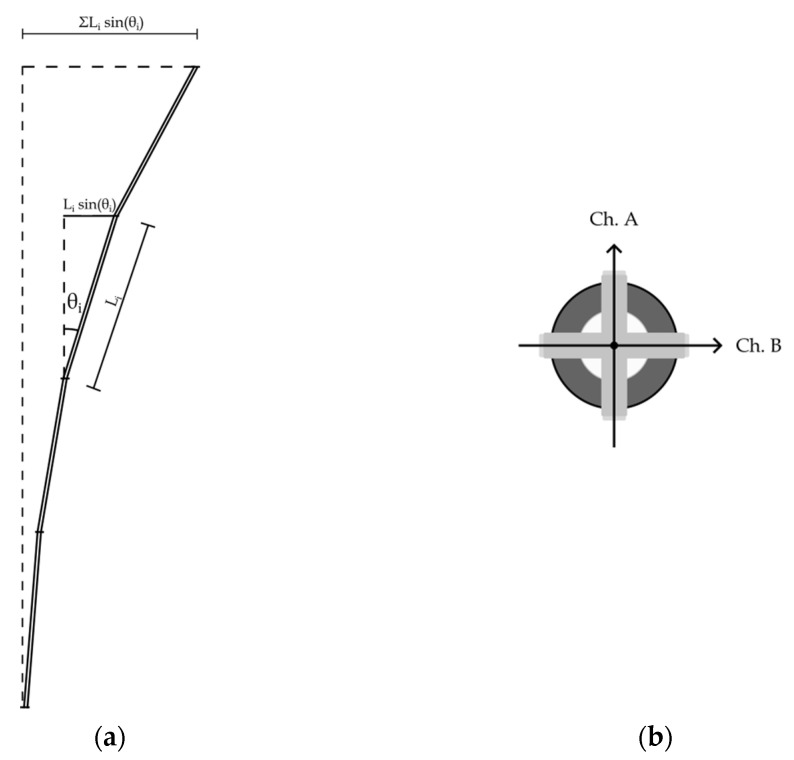
(**a**) Qualitative example of a deformed configuration of the instrument chain. (**b**) Channel alignment of the MEMS sensor.

As displacements are imposed via the usage of the actuators, the IPI within the tube casing deforms accordingly. Each MEMS sensor tracks its relative inclination θ_i_ with respect to the vertical direction, derived by acceleration measurements. The in-plane displacement alignment of the MEMS sensors is reported in Figure 11b. Since the length of each instrument L_i_ is known, it is therefore possible to evaluate the cumulative displacement of the chain as the sum of the individual instrument displacement as ∑L_i_ × sin(θ_i_). This design allows the MD-Profile to provide accurate measurements of the cumulative displacement based on the probes’ angle measurements with respect to the vertical direction [17,20]. A qualitative example of a deformed IPI chain is shown in Figure 11a.

Numerous tests were carried out, which allowed for the revision and redesign of some parts of the measurement apparatus. The presentation will be limited to the most significant tests. The results of the following tests are presented:

- Lab 1: Stability tests on a system chain composed of several probes are performed. The vertical and quasi-vertical configurations have been investigated with chains composed of three to six beams. Presence of drift errors as well as temperature effects on the measures while the instrument is in rest position are investigated.
- Lab 2: The performances of a single beam are investigated. The displacement is imposed on the second extremity of the last beam of the chain. Repeatability is evaluated.
- Lab 3: The performances of the measuring system under the imposed displacement of a node connecting two beams are investigated. This test replicates a net rupture within a structural element such as a fracture. Repeatability is evaluated.
- Lab 4: The performances of the measuring system under the imposed translation of two beams are investigated. This test replicates a discontinuity within a structural element or within soil. Repeatability is evaluated.
- Lab 5: The performances of the measuring system under imposed displacement of the entire chain along a parabolic profile are investigated. This test replicates the deflection of a retaining wall. Repeatability is evaluated.

In Table 1, all the tests are summarized. The main goal of each test, the configurations, and the imposed displacement are reported for each test. In the following tests, the displacements along the A and B directions are parallel and perpendicular to the retaining wall, respectively. The displacement U is imposed in the A direction. For each activity, the most significant results are reported and analyzed. Tests were performed with different initial orientations. The results did not show an appreciable difference as the orientation changed.

## 4. Results

### 4.1. Inclinometer Sensor Calibration

As an example, the results of a beam calibration are reported in Table 2 where the imposed and measured (before and after calibration) values are reported. The calibration process represents a fundamental step for the application of MEMS sensors as the maximum residual error (MRE) decreases from 0.6435% FS pre-calibration to an admissible value of 0.0021% FS post-calibration. The calibration is performed considering hysteresis cycles. A third order polynomial fitting curve is estimated with least square method starting from the measured data. The maximum error is calculated on the whole sensor range.

The MD-Profile is therefore compliant with the standards ISO 18674-3:2017 [36].

The final performances of the sensor are listed:

- Measuring range (bi-axial): ±30° (other ranges available under request)
- Resolution: 0.0002°
- Repeatability: <±0.008°
- Accuracy (Maximum Permitted Error): <±0.025% F.S. (<±0.015°)
- Offset temperature dependency: <±0.01°/°C

### 4.2. Lab 1

First, a stability test has been performed in resting conditions to evaluate possible presence of drift in the measures due to settling movements following the installation of the inclinometric chain. Drift phenomena may be induced by non-negligible stress state induced in the probes or by cable adjustments varying in time. These lead to unpredictable residual displacements. After installation, the beams reached their equilibrium state and accommodated any support displacements, which in this case were assumed to be negligible. The zero measurement was calculated immediately after installation, and no settling period was implemented. Data acquisition was performed every 10 min over a period of one week (approximately 1000 readings~7 days, 144 readings per day).

**Figure 12:**
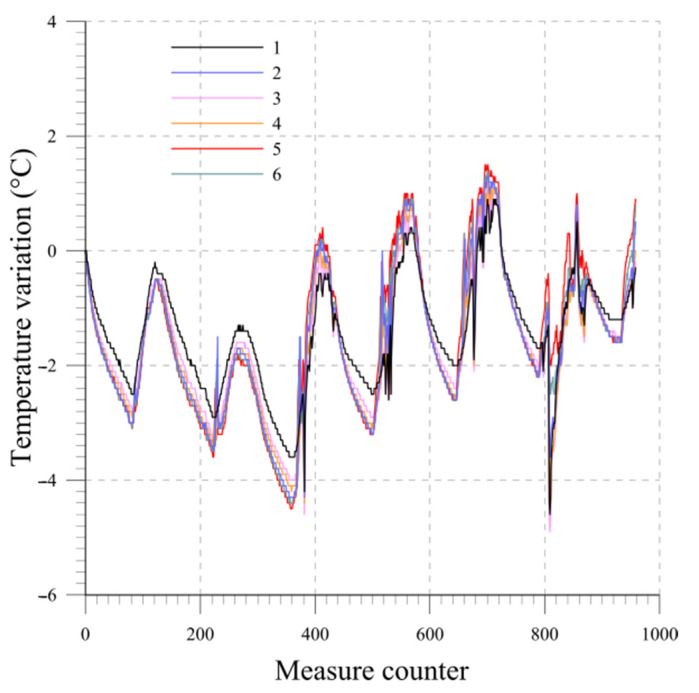
Lab 1: temperature variation readings at equilibrium state.

**Figure 13:**
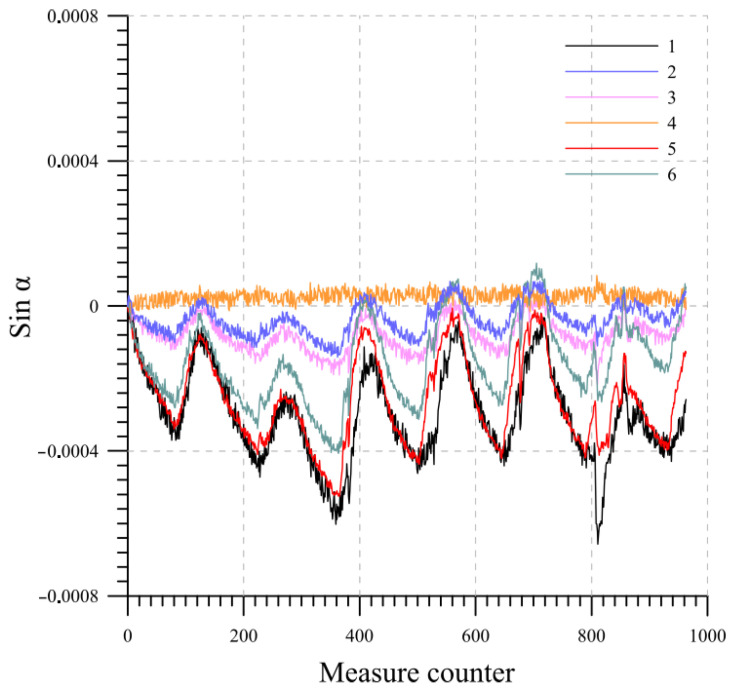
Lab 1: direction A variation readings at equilibrium state.

**Figure 14:**
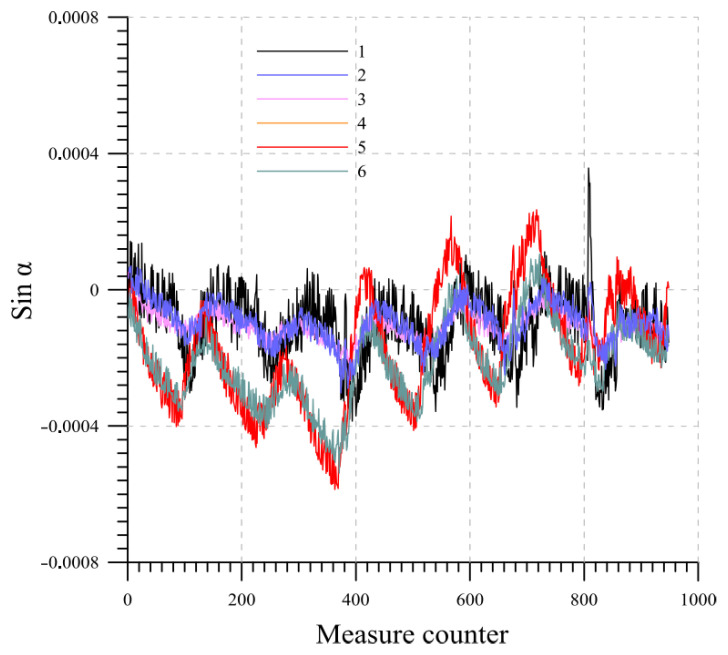
Lab 1: direction B variation readings at equilibrium state.

**Figure 15:**
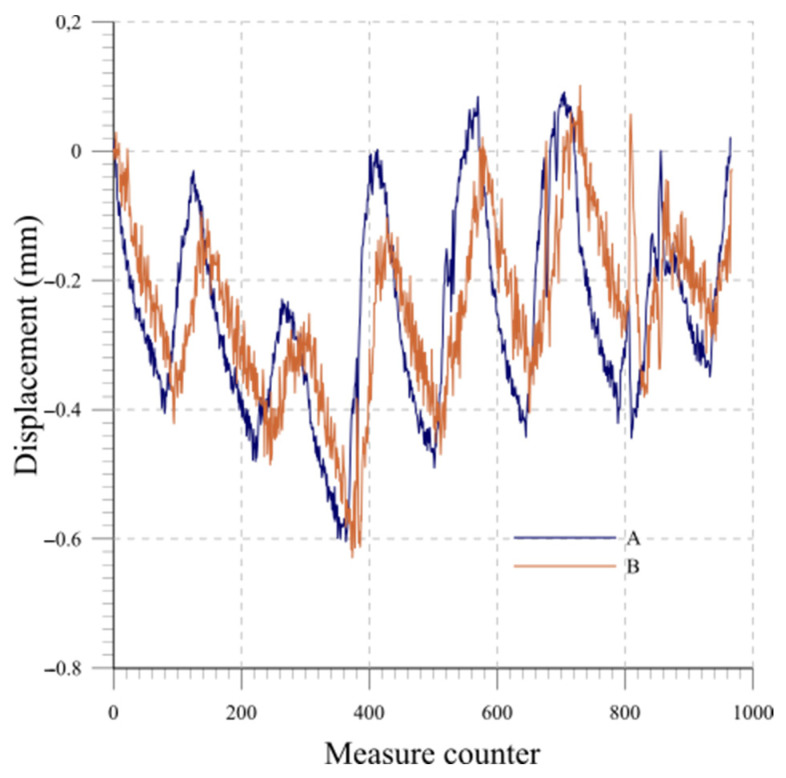
Lab 1: Cumulative displacement variation at equilibrium state: directions A and B.

As an example, the results are presented for a system consisting of six probes, each measuring 0.5 m in length. The probes, thus simulating a real installation, are inserted from the top into a section of inclinometric tube that is 3 m long. The beams are numbered from 1 to 6, starting from the bottom of the chain. The temperature variation measured over the one-week period by each probe at equilibrium state is plotted in Figure 12. Figure 13 and Figure 14 display the readings of the six beams along the two directions A and B while Figure 15 reports the cumulative displacement variation for each channel over the same measured period. The chain behavior exhibited a highly cyclic pattern, with nearly identical readings observed upon the restoration of similar ambient conditions. It is worth noting that a slight alteration in the measurements of probe 1 occurred due to a minor impact on the tube, as evidenced by reading 832.

### 4.3. Lab 2

**Figure 16:**
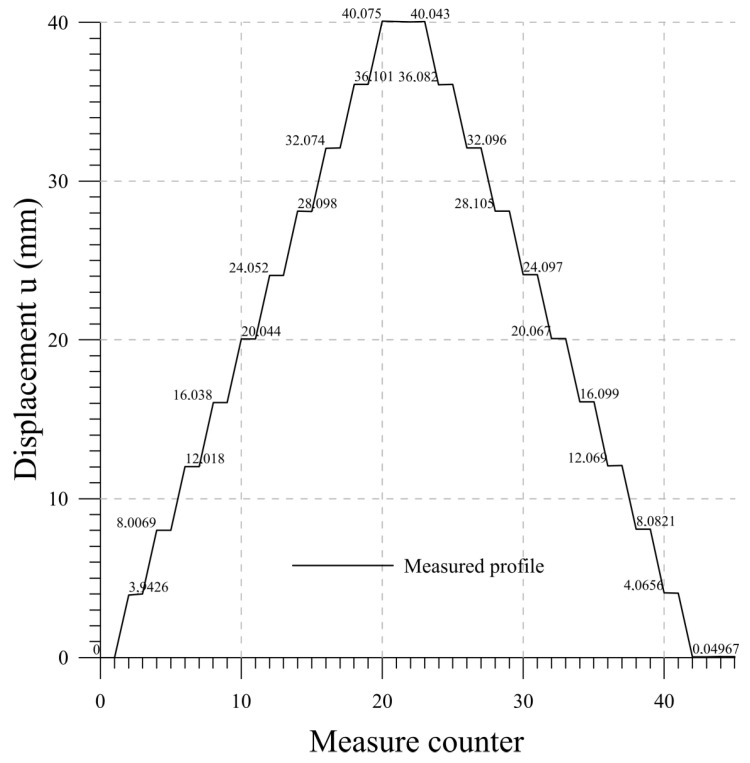
Lab 2: Measurements.

The performance of a single beam is investigated and determined. The free extremity of a beam is moved. This test verifies the correspondence between the imposed displacements and what is determined by the probe configuration estimating the accuracy of the sensor calibration, the effectiveness of the centering device, and the mechanical components. In Figure 16, the measures obtained in a ±40 mm imposed displacement cycle are reported. Measures determined at intermediate positions are also reported. Table 3 illustrates the results of a repeatability test determined on a 10 cycle with an amplitude of ±40 mm.

Several measurement cycles were carried out to test not only the accuracy but also the repeatability of the system and to determine the possible influence on the adjacent rods. The results show high accuracy, good repeatability, and minimum disturbance with the adjacent beams. The performances are summarized in the final Table 4.

### 4.4. Lab 3

**Figure 17:**
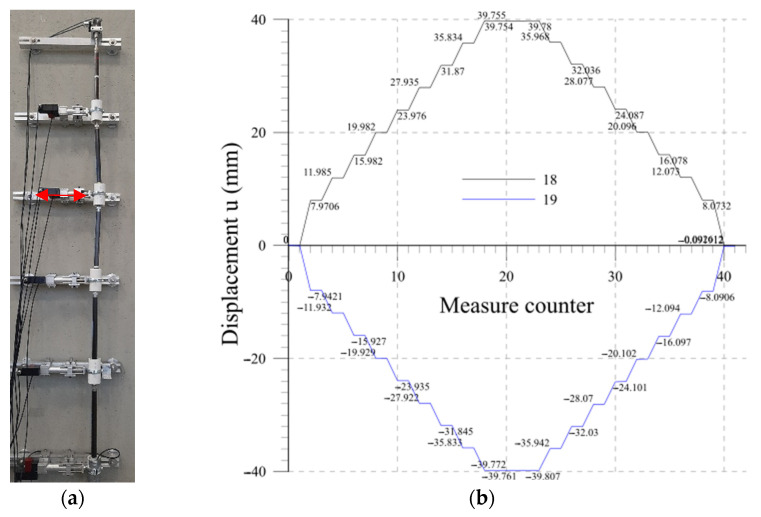
Lab 3: (**a**) setup configuration; (**b**) measurements.

The ability of the measurement chain to detect localized displacements was tested by examining its performance when a node connecting two beams was subjected to an imposed displacement, as illustrated in Figure 17a. This test aimed to simulate a fracture within a structural element. The results, depicted in Figure 17b, show the measured displacement values for the two connected beams during the test obtained in a ±40 mm cycle showing good accuracy and repeatability. A slight shift in the zero configuration of the two probes is observed due to residual interactions between the instruments. The results of a repeatability test are summarized in the final Table 4.

### 4.5. Lab 4

**Figure 18:**
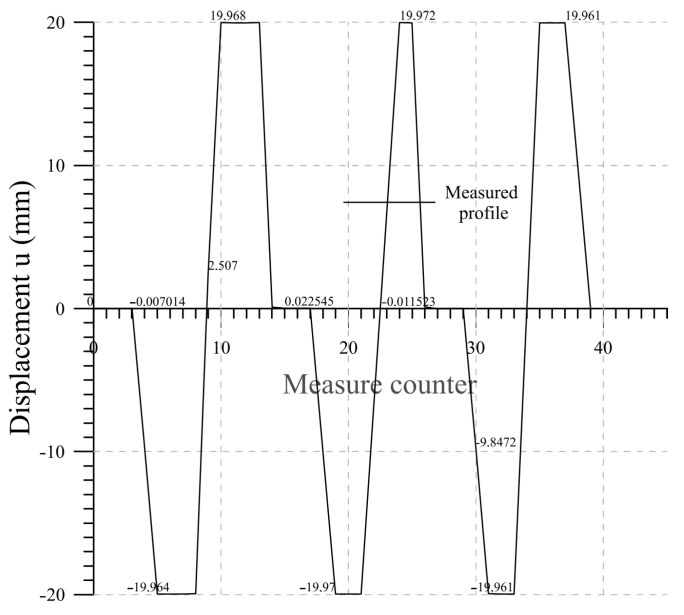
Lab 4: displacement measurements.

The performance of the chain under imposed translation of two beams, mimicking a shear discontinuity within the soil, is investigated. This test is representative of landslide movement. The results are summarized in Figure 18 where the measured displacement values for the rotated beam under a cyclical test with an imposed displacement equal to ±20 mm are plotted. Good performances are achieved as the measured data recover the imposed displacement. Minimal disturbance between each displacement cycle is observed. The performances are summarized in the final Table 4.

### 4.6. Lab 5

**Figure 19:**
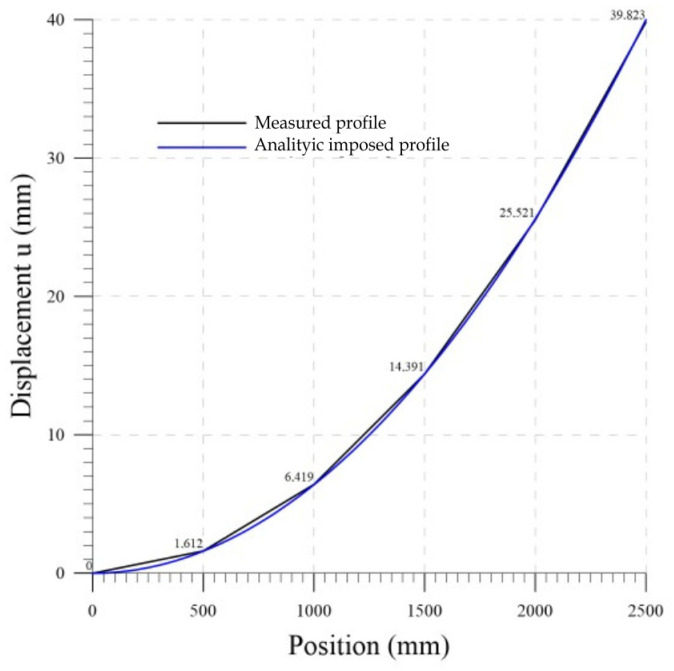
Lab 5: displacement measurements.

Finally, a parabolic profile is applied to the chain. This test replicates the deflection of a retaining wall. The configuration obtained from the probe measures is compared with the imposed profile in Figure 19. The exact values of the imposed displacement are 0, 1.6, 6.4, 14.4, 25.6, and 40 mm at chain positions 0, 500, 1000, 1500, 2000, and 2500 mm, respectively. This test confirms the precision of the measuring system obtained in the previous tests. The performances are summarized in the final Table 4.

## 5. Discussion

In Table 4, some representative results are summarized for the test campaign previously illustrated. For each test the following quantities are reported:

- Measure range: maximum value of the imposed displacement;
- Max absolute error: maximum error recorded in the entire chain;
- Max repeatability error: maximum repeatability error recorded in the entire chain obtained in 10 measures;
- Disturbance of static beams: maximum ghost measure detected by beams in static condition (that should measure null displacement);
- Absolute error on cumulative displacement: maximum error on cumulative displacement.

The developed centering device permits the measuring system to not present a preferred direction of movement. Therefore, the results obtained are to be understood in an arbitrary direction. In fact, the measurement tests were conducted in directions parallel to channels A and B, see Figure 11b, and with predetermined inclinations with respect to the vertical direction of 30° and 60°.

Starting from the results obtained from Lab 1, it is possible to observe absence of drift evidence during the tests, suggesting that a longer test duration than the one examined would not provide substantial value in assessing the long-term stability of the electronic and mechanical components of the measurement system. The temperature conditions at the beginning and end of the test are similar. The cumulative displacement plot is characterized by a cyclical path with a negligible value at the end of the test and directly correlated with the temperature. In real applications, a simple post-processing procedure can be implemented to remove the measurement variation due to temperature and calculate the actual displacement. In fact, the correlation coefficient between the temperature and the displacement varies between 0.95 and 0.99. The system shows no residual displacements; there are no adjustments due to stress states such as inducing system bending and macroscopic viscous phenomena.

Lab 2 investigated precision of a single beam and the possible effects on adjacent rods; the accuracy and repeatability of measures of the system is investigated by imposing a displacement on the last node of the chain. Several measurement cycles were carried out showing high accuracy, good repeatability, and minimum disturbance with the adjacent beams. The absence of direct interaction between the rods is crucial; in fact, the displacement of a node should in no way alter the entire measurement chain.

Lab 3 investigated the accuracy and repeatability of the measures of the system to monitor movement of a single node in the middle of the chain (i.e., net rupture of a structural element). Although the system’s performance was slightly worse than that of the single beam, good accuracy and repeatability were achieved. This configuration, however, is particularly challenging for the system, as it creates a stabilizing moment that compresses the springs on the inner side of the chain. This phenomenon is also evident in the beams that are not directly moved, which experienced a slight shift in the zero configuration of the two probes due to the disturbance.

Lab 4 analyzed the capability of the system to accurately measure movements of soils’ layers or discontinuity within a structural element. High precision is observed and the disturbance of the other probes is minimal, as confirmed by the fact that the zero position is recovered after each loading cycle. Measure repeatability is also noticeable.

Lastly, displacement of the whole chain simulating a slope movement or a retaining wall deformed configuration has been analyzed in Lab 5, highlighting the accuracy and reliability of the system if a parabolic profile is imposed on the chain. In this case, all the probes are involved in the tests.

## 6. Conclusions

This paper described the validation process of an inclinometer chain for use in structural or geotechnical applications.

The main new features of the system are as follows:

- The probes are connected by Cardan joints that allow relative rotations without altering the orientation of the inclinometer chain;
- The centering device allows the probe to remain in the center of the tube;
- The centering device creates a perfect kinematic chain in which the hinges are exactly positioned at the ends of the beams and the interaction between the beams and the tube coincides with the hinges;
- It is possible to use the system in tubes with or without grooves as no preferred measuring orientation is present.

After sensor calibration, validation was performed through the development and use of a test setup that allowed the reproduction of all the steps involved in an inclinometric chain in its entirety. In fact, it is different to investigate the behavior of a single component compared to analyzing the operation of an entire chain. The interaction between the various elements that make up the chain and the interaction with the inclinometric tube induce complications that lead to a deterioration in the performance of the individual probes. Furthermore, it has been possible to investigate the disturbance caused by a localized displacement on the probes that remain in stationary condition. To the authors’ knowledge, this is the first time that an inclinometer chain has been tested so extensively in its entirety by replicating possible application scenarios. The obtained results demonstrated the necessity of validating a measurement chain by analyzing its overall behavior and not limiting the study on the performances of a single element. Furthermore, this study allowed effective solutions to be identified during the development of the instrumentation that could not have been conceived and tested otherwise.

An ad hoc test with a redundant measurement system, similar to what was proposed in [30], on an in situ installation is being designed and implemented. In particular, a classical application cannot provide consistent information to validate the operation of the instrumentation. In fact, in addition to the difficulties of chain insertion, which have already been investigated in the laboratory on a 12-element chain, only the displacement of the top of the chain can be controlled by remote measurement techniques with lower accuracies and resolutions than those of the system. The system cannot be considered to be in a stable condition but is subject to continuous displacements; therefore, the local measurement obtained from the chain can only be validated when compared with another local measurement.

In the future, information will be collected from various applications in the field, including deep excavations and diaphragm walls, retaining walls, landslides, and unstable slopes, and post-processing algorithms based on artificial intelligence will be implemented.

## Figures and Tables

**Table 1:** Summary of the performed tests.

Activity	Goal	Scheme	Imposed Displacement
Lab 1	Determination of presence of drift in the measurements in rest conditions after installation	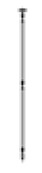	U = 0 mm, α = 0 mm
Lab 2	Determination of the performance of a single beam	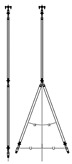	U = ±40 mm, α = ±4.5885°
Lab 3	Determination of the performance of the chain under imposed displacement of a node connecting two beams (fracture simulation)	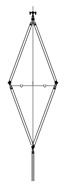	U = ±8, 12, 16, 20, 24, 28, 32, 36, 40 mm
Lab 4	Determination of the performance of the chain under imposed translation of two beams (discontinuity simulation)	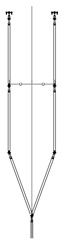	U = ±8, 12, 16, 20, 24, 28, 32, 36, 40 mm
Lab 5	Determination of the performance of the chain under imposed displacement of the entire chain along parabolic profile (wall deflection)	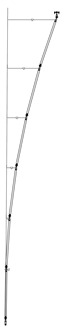	U = ±40 mm at the top

**Table 2:** Comparison between the imposed value and the sensor measurements before and after the calibration process. Additional information can be found at https://sisgeo.com/wp-content/uploads/Prodotti/IPI%20In-Place%20Inclinometers/MD-Profile%20array/Inglese/Datasheet/MD-PROFILE_system_EN_02.pdf (accessed on 30 July 2023).

	Ch. A	
Imposed Value [sin(α)]	Pre-Calibration [sin(α)]	Post-Calibration [sin(α)]
0.498826	0.462448	0.498822
0.340494	0.308479	0.340494
0.171797	0.145000	0.171787
−0.001974	−0.022876	−0.001979
−0.175698	−0.190273	−0.175694
−0.344155	−0.352245	−0.34417
−0.502041	−0.503681	−0.502046
−0.501978	−0.503609	−0.50197
−0.343707	−0.351799	−0.343705
−0.174904	−0.1895031	−0.174894
−0.000913	−0.021847	−0.000912
0.172825	0.146008	0.172828
0.341354	0.309319	0.341359
0.499126	0.462745	0.499128
**MRE [%FS]**	0.6435%	0.0021%

**Table 3:** Lab 2: Repeatability test for +40 mm.

Measures (mm)										Mean (mm)	St. Dev
40.035	40.043	40.033	40.047	40.049	40.041	40.032	40.039	40.023	40.047	40.036	0.0078

**Table 4:** Summary of the system performances.

Test	Range (mm)	Max Absolute Error (mm)	Repeatability (mm)	Disturbance of Static Beams (mm)	Absolute Error on Cumulative Displacement (mm)
Lab 1	null	<0.0001 rad each beam	<0.0001 rad each beam	Not available	±0.12 mm
Lab 2	±40 mm	±0.1 mm	±0.05 mm	±0.05 mm	Not available
Lab 3	±20 mm	±0.1 mm	±0.05 mm	±0.05 mm	±0.05 mm
Lab 3	±40 mm	±0.25 mm	±0.1 mm	±0.1 mm	±0.1 mm
Lab 4	±20 mm	±0.08 mm	±0.04 mm	±0.05 mm	±0.08 mm
Lab 5	±20 mm	±0.1 mm (cumulative displacement)	±0.05 mm	Not available	±0.1 mm
Lab 5	±40 mm	±0.25 mm (cumulative displacement)	±0.1 mm	Not available	±0.25 mm

## Data Availability

Data availability on the sensors can be requested to SISGEO SRL.
